# Clinical Evidence of Vestibular Dysregulation in Colicky Babies Before and After Chiropractic Treatment vs. Non-colicky Babies

**DOI:** 10.3389/fped.2021.668457

**Published:** 2021-05-28

**Authors:** Jan Hoeve

**Affiliations:** Chiropractie Staphorst, Staphorst, Netherlands

**Keywords:** infantile colic, vestibular, dysregulation, clinical index, sensory neuromodulation

## Abstract

**Background:** To date, after 65 years of research that was primarily directed at differentiating between normal and colicky crying, the cause of infantile colic remains elusive and no definitive cure has been found. Given the general absence of pathology, colicky crying is widely considered the extreme end of a spectrum of normal crying behavior. However, evidence gleaned from scattered sources throughout the literature suggests that infantile colic may be the behavioral expression of physiological brainstem dysregulation, particularly of the vestibular and autonomic systems. The purpose of this study is to present a five-point clinical index of vestibular (hyper) activity and its application to investigate vestibular dysregulation in colicky and non-colicky babies.

**Methods:** One hundred and twenty consecutive colicky babies were evaluated using this index, before and after a very gentle vibratory treatment, and compared to 117 non-colicky babies.

**Results:** Before treatment, of 120 colicky babies only 2 (1.7%) scored 0, whereas 118 (98.3%) scored 1–5. Of 117 non-colicky babies 89 (76.1%) scored 0 and 28 (23.9%) scored 1–3, none scored 4–5. The odds ratio is OR (CI 95%) 187.54 (43.52–808.09). After treatment 111 (92.5%) scored 0 and 9 (7.5%) scored 1–3, none scored 4–5. A McNemar test showed the difference before and after to be significant (χ^2^ = 109.00, *p* < 0.001). For colicky babies the mean vestibular score is 2.88 (SD 1.22), compared to 0.37 (SD 0.73) for non-colicky babies, a difference of 87.2%. After treatment the score decreased from 2.88 (SD 1.12) to 0.10 (SD 0.40), or 96.5%.

**Conclusion:** Colicky babies are not just infants who cry a lot. They also show clinical evidence of vestibular dysregulation. Treatment aimed at relaxing tight sub-occipital musculature by means of gentle vibrational stimulation may be effective in decreasing vestibular hyperactivity, signifying an improvement in brainstem regulation. The vestibular index opens the prospect for development into a tool toward an objective and practical clinical diagnosis of infantile colic.

## Introduction

Infantile colic is defined as excessive and inconsolable crying and fussing in an otherwise healthy and well-fed infant who is > 5 months old. It is considered a benign behavioral disorder with a prevalence of 15–25% that spontaneously resolves after 4–5 months (1). The diagnosis is commonly based on the (modified) Wessel criteria of crying for at least 3 h a day during at least 3 days in the preceding week (1, 2). At present, after 65 years and a great deal of research that was primarily focused on the excessive crying *per se* the cause of colic remains elusive and no distinct cures have emerged. The one thing that has become clear, however, is that usually there is no pathology (3).

Babies function at a basic level of visceral brainstem reflexes, because higher inhibitory modulating structures are not sufficiently developed yet (4). This means that any dysregulation present may be expected to arise from this same level. Although little researched, symptoms that are commonly associated with colic can be found scattered throughout the literature. These include: (1) asymmetric posture (C-curve) and head preference (5) even while asleep, which may lead to developmental plagio/brachiocephaly; (2) extensor hypertonicity and pseudo-opisthotonic posture (5, 6); (3) upper-cervical movement/joint dysfunction, muscular tightness and occipital tenderness (7); (4) high levels of stress and stress arousal (6); (5) breastfeeding difficulties (7–10); (6) gastro-intestinal disorders such as regurgitation /Ger(d) and intestinal cramps with or without obstipation or dyschesia (11). Taken together the associated symptoms may point to dysregulation of the vestibular and autonomic systems. Yet, the possibility that infantile colic may be the behavioral expression of underlying physiological dysregulation at the brainstem level, has remained largely unexplored.

This present study deals with a clinical assessment of the vestibular system. A five-point clinical index of vestibular (hyper) activity is presented and applied as a tool to evaluate brainstem dysregulation in colicky babies before and after chiropractic treatment compared to non-colicky babies.

## Clinical Index of Vestibular (Hyper) Activity

The index was developed in our chiropractic clinic where it has been utilized for over 3 years. Mild rhythmic vestibular stimulation is known to have a relaxing, soothing effect (12), whereas vestibular overstimulation tends to be uncomfortable and may lead to dizziness, nausea or even vomiting (13). Therefore, if a baby does not react well to mild vestibular stimulation this could be taken as a possible sign of vestibular hyperactivity.

Our clinical index is comprised of five items, each consisting of a statement that can be answered by a simple agree/disagree. Each “agree” earns one point and each “disagree” earns zero points. So, the babies may have a score between 0 and 5. The statements are based on literature reports, personal observations and reported parental experiences:

*(A). Your baby does not calm down or fall asleep during a car ride*. It is a common practice all over the world that babies are rocked in a cradle or in a carrying sling to help them calm down and fall asleep. Riding in a car may have a similar soothing effect as non-colicky babies generally fall asleep during a car trip. Some desperate parents take their colicky baby on evening car rides in the hope that they may stop screaming. In many cases this seems to work, but not always as available research is inconclusive. Some parents report that the baby relaxes only while the car is moving, but that with every stop at a traffic light the screaming starts again. This suggests that motion and not just car vibration or white noise is important to induce a calming effect. If the baby does not calm down or fall asleep during a car ride this is taken as a possible sign of vestibular hyperactivity.

*(B). Your baby does not calm down or fall asleep when held against your chest or cradled in the crook of your arm, while you are walking around at a brisk pace*. When a mouse or a cat mother wants to move her young she takes them in her mouth and carries them to the new spot. The little pups react with a mammalian calming reflex that is characterized by going limp, by a decrease in ultrasonic vocalizations (in mice) and a decrease in heart rate. These changes are mediated by the parasympathetic system and the cerebellum and in mice are dependent on tactile and proprioceptive stimuli (14). Human babies show a similar calming response to carrying i.e., a decrease in voluntary movement, heart rate and crying (14). However, the observation that the response is much stronger when the mother is walking at a brisk pace than when she is just sitting and holding the baby suggest that vestibular stimulation is at least equally important. When asked, many mothers of colicky babies deny that their infant cries excessively (for nobody wants a colicky baby or a cry baby). Upon further questioning they may tell that they have to carry their baby and walk around almost all day and that, if they do not, the baby will cry and be restless. This agrees with Esposito's (14) observation that the calming response stops the moment the carrying ends. If the baby does not calm down when held against the mother's chest while she is walking at a brisk pace this is taken as another possible sign of vestibular hyperactivity.

*(C). When your baby has fallen asleep against your chest you cannot lay the baby supine in the crib without the baby waking up and crying*. This item also relates to item (E). A common observation by parents is that colicky babies are not comfortable lying on their back. When held upright against the parent's chest, particularly if the parent is leaning backwards, the baby may be relatively calm or even fall asleep, but as soon as the baby is even gently laid down the screaming starts within a few minutes (without showing the characteristics of the Moro reflex). Some parents resort to spending the night sitting partially upright in bed with the baby sleeping against their chest. If the sleeping baby cannot be laid down supine in a crib without waking up this is taken as a sign of possible vestibular hyperactivity, because the only difference between the two positions is the orientation with respect to gravity.

*(D). When sleeping the baby may wake up with a scream, showing the symptoms of the Moro reflex*. Many parents report that once their colicky baby is asleep the infant may suddenly wake up with a scream, showing the characteristics of the Moro reflex. Since this behavior is not induced by an external stimulus such as a loud noise and because the Moro reflex, in contrast to the Startle reflex, is essentially a vestibular reflex (15), this behavior is taken as a possible sign of vestibular hyperactivity. Some parents report that when carrying the baby on their arm while walking down the stairs the baby will scream with every descending step. Other mothers describe that the baby will scream the moment they sit down on a chair in preparation for breastfeeding. Such observations suggest that in these babies the vestibular threshold to the Moro reflex may be set low. It is surmised that while asleep the baby may have a sudden falling sensation as if the floor is dropping away from underneath them.

*(E). The baby is much more comfortable lying inclined in a car seat than supine in a crib*. This item also relates to item (C). Many parents report that the baby is much more relaxed and sleeps much better when lying inclined in a car seat (Maxi-Cosi) than supine in a crib or bed. This observation may also be a sign of vestibular hyperactivity, because when inclined at an angle of some 30 degrees the position of the lateral semicircular canal is approximately vertical and presumably less receptive to stimuli (16). The other relatively stable position is at a forward tilt of some 30 degrees against the parents chest when the lateral canal is approximately horizontal.

## Methods

This study is based upon a convenience sample of colicky and non-colicky babies. Included were 120 consecutive, unhappy babies [mean age 6.4 (SD 4.64) weeks; range 1–35 weeks; male 68.3%; female 31.7%], who presented to our chiropractic clinic for infantile colic and were treated with a very gentle method aimed at relaxing tight occipital/upper-cervical musculature. Excluded were 5 babies who withdrew early from treatment for financial reason because they were not covered by health insurance. All fulfilled the modified Wessel criteria (1) of crying 3 h a day for 3 days during the preceding week. The actual treatment involves the application of slight, pulsed vibrations at selected points in the occipital/upper-cervical region by means of a spring-loaded reflex device (J-Tech device, zero setting, tangentially applied; no impact, just slight vibrations), aimed at relaxing tight sub-occipital musculature. This device and similar ones are widely used in the chiropractic treatment of musculo-skeletal conditions, albeit at a higher instrument setting. The use of a somewhat different vibrotactile device was reported in a medical study investigating the therapeutic effects of parasympathetic neuromodulation in rheumatoid arthritis patients by means of cutaneous sensory stimulation applied to the cymba of the external ear (17). In the present study two treatments per week were given for an average of 2 weeks i.e., an average of four treatments in all (Table 1). The treatment was terminated when (1) the parents reported satisfactory improvement in general well-being, the infant having changed from a highly stressed, screaming baby into a relaxed, happy child; and (2) the treating chiropractor was satisfied that tight occipital/upper-cervical musculature had relaxed. As controls 117 non-colicky, happy babies [mean age 7.5 (SD 3.72) weeks; range 3–22 weeks; male 47%; female 53%] were included, who presented to our clinic as part of an ongoing program offering free check-ups. All colicky babies were categorized in four age groups: 1–4, 5–8, 9–12 weeks, > 13 weeks (Table 1).

**Table 1:** Characteristics colicky vs. non-colicky babies.

	**Colic** ***n =* 120**		**Non-colic** ***n =* 117**
Age	6.37 (4.84)		7.45 (3.72)
Male	68.3%		47.0%
Female	31.7%		53.0%
**Total vestibular score**			
	**Colic Before**	**Colic After**	**Non-colic**
Score = 0, n (%)	02 (01.67)	112 (93.33)	89 (76.07)
Score = 1, n (%)	10 (08.33)	6 (5.00)	15 (12.82)
Score = 2, n (%)	31 (25.83)	1 (0.83)	11 (9.40)
Score = 3, n (%)	47 (39.17)	1 (0.83)	2 (1.71)
Score = 4, n (%)	19 (15.83)	0 (0)	0 (0)
score = 5, n (%)	11 (09.17)	0 (0)	0 (0)
Mean (SD)	2.88 (01.12)	0.10 (0.40)	0.37 (0.73)
**Vestibular score by individual characteristics**			
	**Colic Before**	**Colic After**	**Non-colic**
A—Car ride, n (%)	34 (28.33)	0 (0)	7 (0.06)
B—Carrying, n (%)	48 (40.00)	0 (0)	1 (0.85)
C—Laying down, n (%)	97 (80.83)	2 (1.66)	14 (11.97)
D—Moro, n (%)	96 (80.00)	4 (3.33)	12 (10.26)
E—Car seat, n (%)	71 (58.33)	1 (0.83)	9 (7.69)
**Total Vestibular score by age group**			
	**Colic before**	**Colic after**	**% decrease**
1–4 weeks, Mean (SD)	2.70 (1.09)	0.06 (0.23)	97.8
5–8 weeks, Mean (SD)	3.10 (1.10)	0.10 (0.38)	96.8
9–12 weeks, Mean (SD)	2.76 (1.15)	0.18 (0.72)	93.5
> 13 weeks, Mean (SD)	3.00 (1.27)	0.18 (0.40)	94.0
**Number of treatments**			
Overall	4.45 (0.89)		
1–4 weeks, Mean (SD)	4.40 (0.98)		
5–8 weeks, Mean (SD)	4.59 (0.91)		
9–12 weeks, Mean (SD)	4.53 (0.62)		
> 13 weeks, Mean (SD)	4.09 (0.70)		
Odds Ratio colic/non-colic	187.54 (43.52–808.09)		
***χ***^**2**^ before/after	109.00 *p <* 0.001		

**Figure 1:**
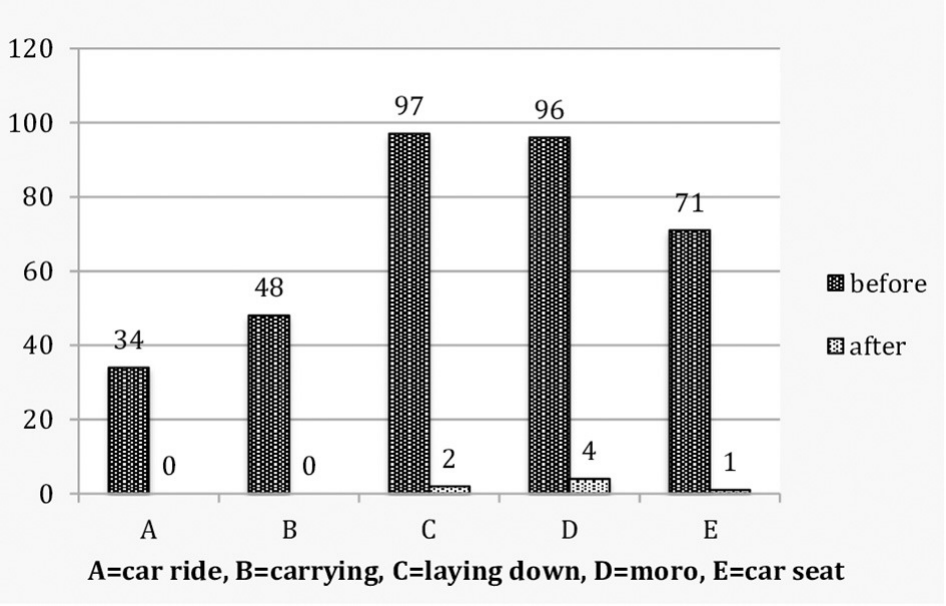
Colicky babies: Vestibular score by individual characteristics before and after treatment.

**Figure 2:**
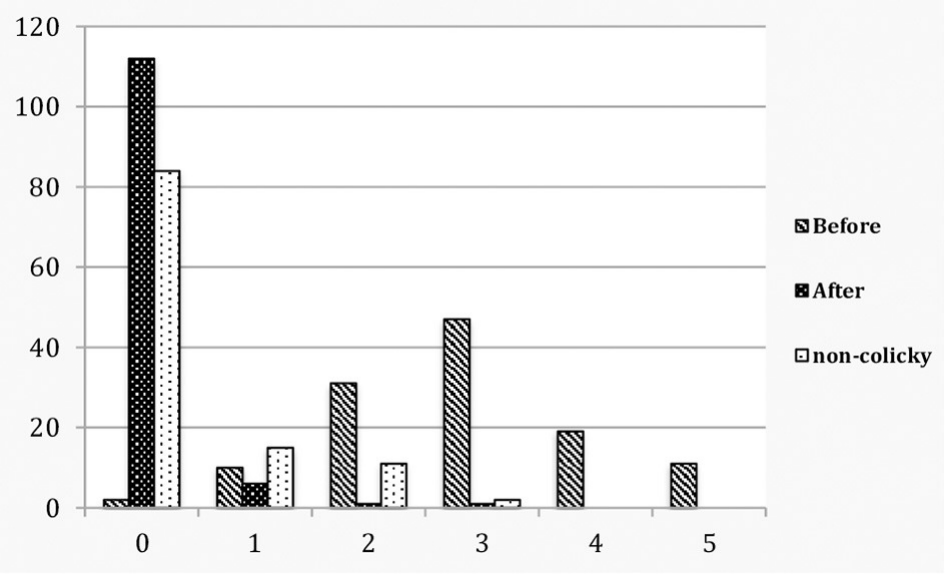
Colicky babies before and after treatment compared to non-colicky babies: total vestibular score.

Colicky babies, before and after treatment, and non-colicky babies were evaluated for vestibular dysregulation by means of the above clinical index of vestibular (hyper) activity (Table 1; Figures 1, 2).

### Statistical Analysis

The vestibular scores are given as n (%) and as Means with SD (Table 1). Binomial logistic regression analysis was used to study the association between vestibular scores (1–5) of colicky and non-colicky babies. The results are given as an odds ratio (OR) with 95% CI (Table 1). A McNemar test was used to study differences in vestibular score comparing colicky babies before and after treatment. Statistical computations were made using SPSS.

## Results

Before treatment, of the 120 colicky babies only 2 (1.7%) scored 0, whereas 118 (98.3%) scored 1–5. In the control group of 117 non-colicky babies 89 (76.1%) scored 0, none scored 4–5 and 28 (23.9%) scored 1–3. The odds ratio is OR (CI 95%) 187.54 (43.52–808.09). After treatment 111 (92.5%) scored 0, none scored 4–5 and 9 (7.5%) scored 1–3 (Table 1; Figures 1, 2). A McNemar test showed the difference before and after treatment to be significant (χ^2^ = 109.00, *p* < 0.001). Before treatment colicky babies had a mean vestibular score of 2.88 (1.12) compared to 0.37 (0.73) for non-colicky babies, a difference of 87.2%. After treatment the score had decreased from 2.88 (1.22) to 0.10 (0.40), a drop of 96.5%. The number of treatments for each age group were very similar and close the number of treatments overall [4.45 (0.89)]. The decrease of the vestibular scores for each age group were very similar and close to the 96.5% decrease overall (Table 1).

## Discussion

Much research in infantile colic has focused on the excessive crying *per se*, as this is, at least for the parents, the most distressing aspect. A recent review established that normal crying in babies < 3 months old averages 2 h per day during the first 2 weeks, increases slightly to 2 h 15 min at week 6 and then decreases to 1 h 10 min by week 12 (1). Nonetheless, there is a wide variation in crying time, some babies cry as little as half an hour a day, whereas others cry for many, many hours. With regard to the cause of colic multiple suggestions have been made, both pathological and non-pathological. These include dietary causes such as cow's milk or fructose intolerance (18), GER(D) (11), neurodevelopmental problems, behavioral problems, difficult temperament, transient hyperresponsivity (6, 19), immaturity of the gut, over/underfeeding (20), imbalance of the autonomic nervous system with high levels of distress and activation of the HPA-axis and the sympathetic-adrenal system (9). None of these have been conclusively proven. In the absence of a distinct cure management strategies emphasize the importance of reassuring the parents and providing social support (21).

Because in infantile colic there generally is no recognizable pathology it is widely suggested that colic is just the extreme end of normal crying behavior (22, 23). However, this is unlikely from an evolutionary as well as an energy perspective. When babies cry they convey a message to the parents that they are not comfortable and that their needs are to be met, be these physiological or emotional (24). Since a baby's crying has a high emotional impact parents find it difficult to ignore (25, 26). Normally this is fine because it compels the parents to tend to the baby's needs, and once these have been met, the crying and fussing stops. From an evolutionary perspective it is unlikely that the baby would engage in non-functional crying, because in order for the system to work the baby should “never cry wolf.” From an energy perspective it is also unlikely because the excessive crying costs up to 20 times more energy compared to the quiet sleeping state (27). That energy would be better spent on growing, rather than wasted on crying for no reason. Other authors have pointed to the dynamics of family relationships and sub-optimal infant-parent interaction as a possible cause of colicky behavior (28). This too is unlikely because it cannot account for the common observation that one member of a pair of twins may be colicky whereas the other is not (personal observations in our clinic). It seems then that instead of accepting the premise that all babies cry, we should consider that babies do not cry unless they have a good reason to do so i.e., signaling discomfort or need and calling upon the parents to look after them. This puts infantile colic in a different perspective. If crying is a graded non-specific signal of distress (29) the question becomes one of why is it that such a high percentage of babies are so thoroughly uncomfortable and in so much distress (15–25%).

The present study reveals that colicky babies are not just infants who cry a lot. They also show clinical evidence of underlying vestibular hyperactivity. The reason for this hyperactivity may be inferred from the realization that the vestibular system is modulated by upper-cervical proprioception and by the visual system (30, 31). In young infants, however, the visual system cannot play a significant role yet as visual gaze and the vestibulo-ocular reflex are still immature (32–34). For this reason vestibular modulation in small babies may primarily depend on upper-cervical proprioception. Afferent proprioceptive input from upper-cervical segments (C1-3) and from axial structures project to the vestibular nuclei and indirectly to the medial cerebellar cortex and its Purkinje cells. As these Purkinje cells are inhibitory to the fastigial output nucleus, they allow the medial cerebellum to exert an inhibitory modulating influence on the vestibular system (35–37). A state of vestibular hyperactivity may ensue from even slight, subclinical, diminished inhibition by the cerebellum (13). Consequently, aberrant proprioceptive traffic into the vestibular system and the medial cerebellum arising from tight sub-occipital musculature, possibly acquired at birth (5), could conceivably provide a mechanism for dysregulation and hyperactivity of the vestibular nuclei. Conversely, treatment aimed at relaxation of the tight musculature may be expected to restore regular proprioceptive flow and facilitate normalization of the inhibitory modulation of the vestibular nuclei by the medial cerebellum.

In the present study gentle relaxation of upper-cervical tight musculature by means of a vibratory method was associated with 96.5% decrease in the vestibular score, indicating a decrease in vestibular hyperactivity and an improvement of vestibular regulation. In the absence of a control group of untreated colicky babies this decrease cannot be conclusively attributed to the treatment that was administered. The observed decrease, however, is unlikely to reflect the natural course of colic as a self-limiting disorder, because for all four age categories the number of treatments required as well as the decrease of the vestibular score turned out to be closely similar and, therefore, not dependent on age (Table 1). Moreover, after 2 weeks of treatment the babies in category 1–4 weeks were 3–6 weeks old. Considering that this is the peak age of infantile colic (1), it is clear that the decrease cannot reflect the natural course of the disorder. Therefore, it seems likely that the documented decrease in vestibular score may derive from the treatment that was applied. If so, this would suggest that central neuromodulation may be accomplished not only by cutaneous vibratory stimulation to cymba of the external ear (16), but also by similar sensory stimulation of suboccipital musculature, at least in babies. Other examples of sensory neuromodulation have been reported in the literature (37).

The vestibular system arises early in embryological development. In concordance with their central role in many regulatory processes the vestibular nuclei have extensive projections to other brainstem nuclei (37). This means that any vestibular dysregulation present may well be propagated to those very same nuclei, particularly of the autonomic system (13), which may conceivably lead to a spectrum of associated symptoms such as listed in the introduction.

Strengths of the present study are that the five-point vestibular index is easily applied in a clinical setting and that a significant 87.2% difference between colicky and non-colicky babies could be documented. In current practice the diagnosis of infantile colic is generally one of exclusion. The vestibular index presented here, if confirmed and validated, could be developed into a tool that allows a clinical diagnosis to be made on the basis of distinct and objective criteria.

A weakness is the absence of a proper control group of untreated colicky babies. However, in a clinical setting where parents present their babies with the intention that they are treated, selection of a control group would be unethical and also impossible to achieve. With regards to future research it is suggested that the direction should be broadened from being almost exclusively focused on the excessive crying *per se*, to also include the symptoms of possible dysregulation at the brainstem level.

## Conclusions

Colicky babies are not just infants who cry a lot. They also show clinical evidence of underlying vestibular dysregulation i.e., dysregulation at the brainstem level. Gentle treatment aimed at correcting upper-cervical muscular tightness by means of vibratory proprioceptive stimulation may be effective in decreasing vestibular hyperactivity, signifying an improvement of brainstem regulation. The clinical vestibular index presented here offers the potential for development into a tool toward an objective and practical diagnosis of infantile colic.

## Data Availability Statement

The raw data supporting the conclusions of this article will be made available by the authors, without undue reservation.

## Ethics Statement

Data were collected as part of regular treatments and check-ups. The data were extracted from patient files. Under Dutch law no approval of an ethics committee is required. All individuals who present at our clinic for treatment or check-up are invited to give their written informed consent (a) for treatment, and/or (b) that a report may be sent to other health professional, (c) that their data may be used anonymously for research purposes and (d) incorporated in scientific publications. For all babies included in the present study parents or guardians gave their written consent.

## Author Contributions

The author confirms being the sole contributor of this work and has approved it for publication.

## Conflict of Interest

The author declares that the research was conducted in the absence of any commercial or financial relationships that could be construed as a potential conflict of interest.
